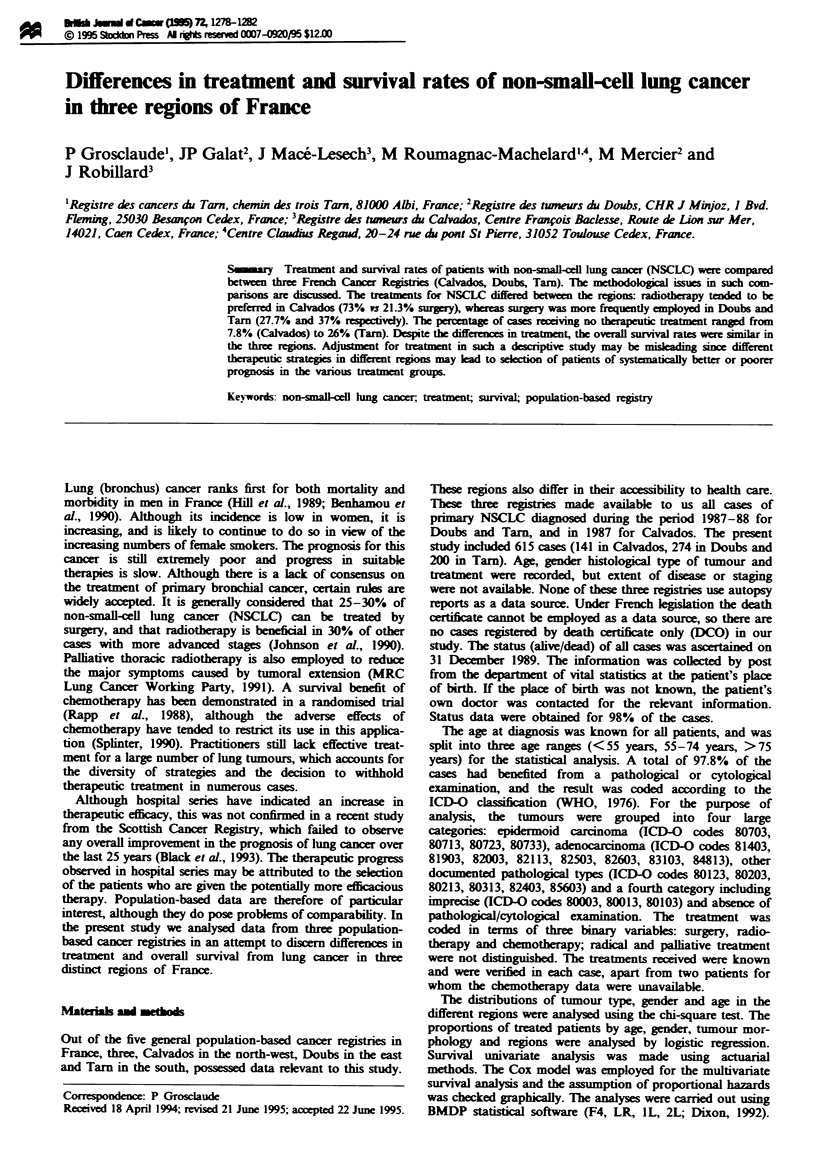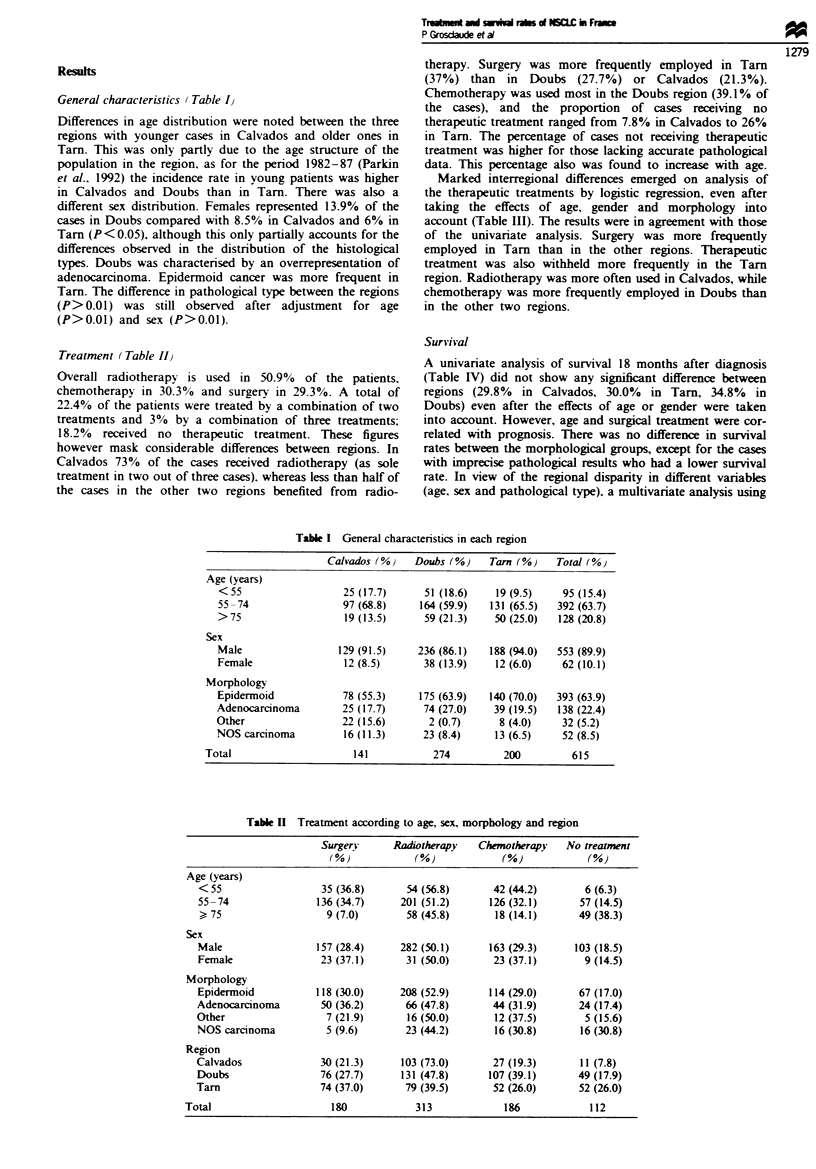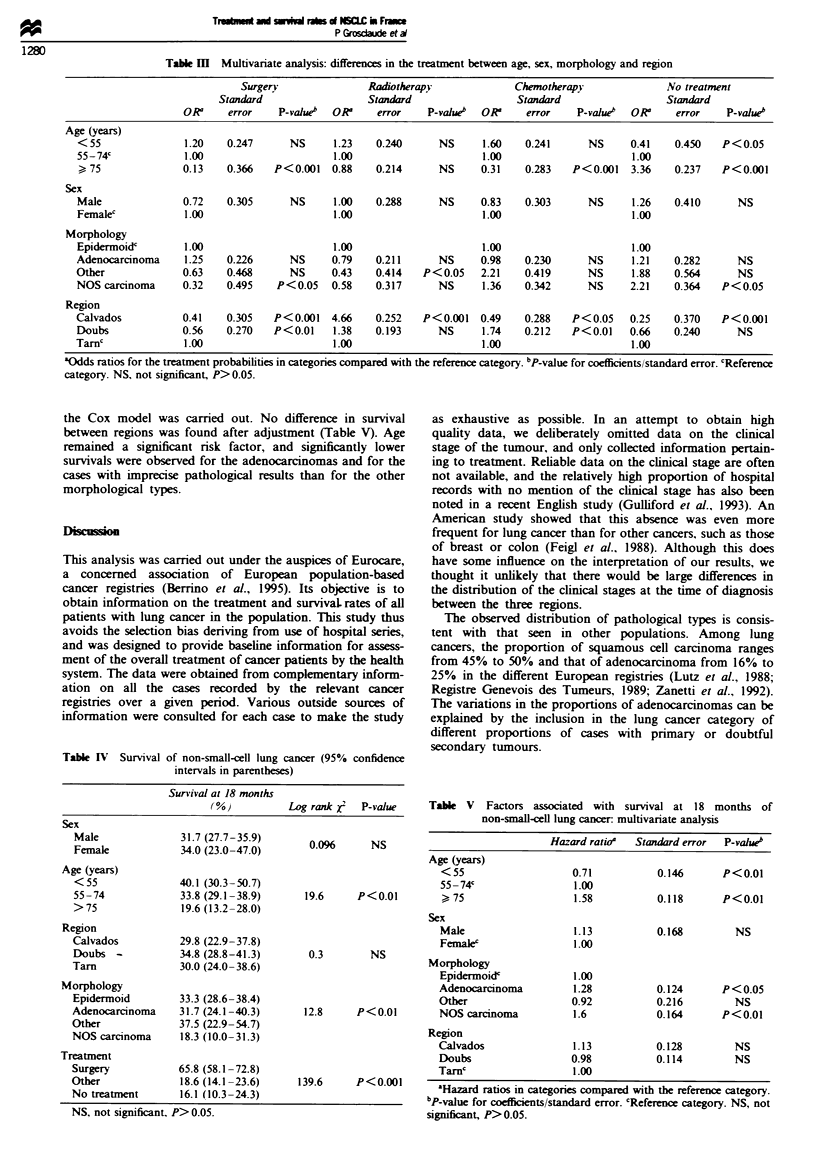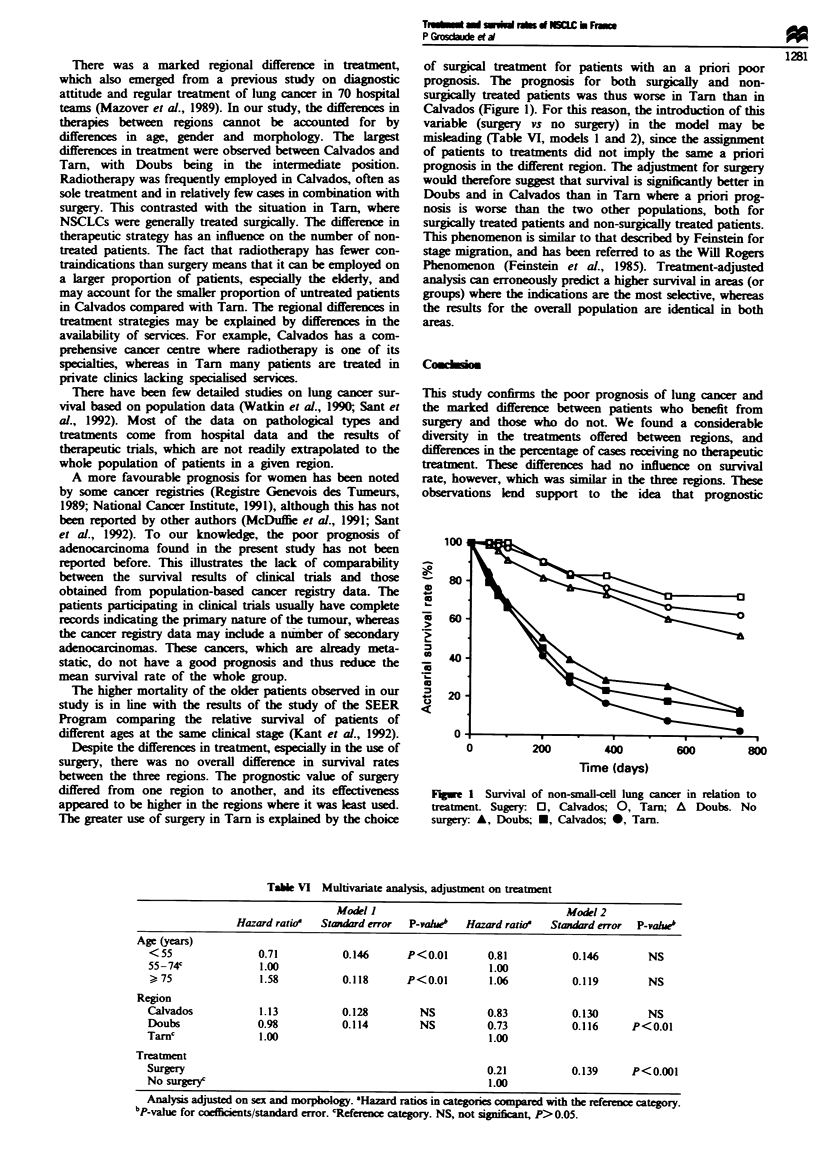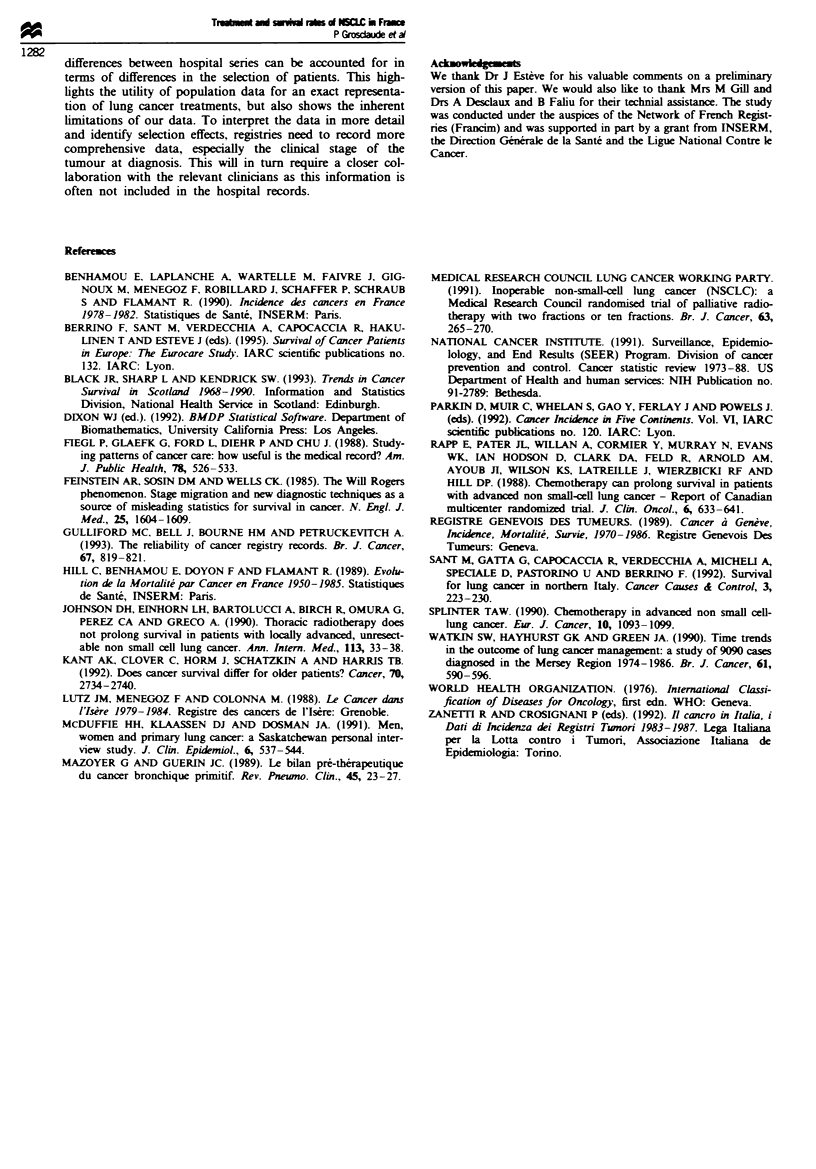# Differences in treatment and survival rates of non-small-cell lung cancer in three regions of France.

**DOI:** 10.1038/bjc.1995.500

**Published:** 1995-11

**Authors:** P. Grosclaude, J. P. Galat, J. Macé-Lesech, M. Roumagnac-Machelard, M. Mercier, J. Robillard

**Affiliations:** Registre des cancers du Tarn, chemin des trois Tarn, Albi, France.

## Abstract

Treatment and survival rates of patients with non-small-cell lung cancer (NSCLC) were compared between three French Cancer Registries (Calvados, Doubs, Tarn). The methodological issues in such comparisons are discussed. The treatments for NSCLC differed between the regions: radiotherapy tended to be preferred in Calvados (73% vs 21.3% surgery), whereas surgery was more frequently employed in Doubs and Tarn (27.7% and 37% respectively). The percentage of cases receiving no therapeutic treatment ranged from 7.8% (Calvados) to 26% (Tarn). Despite the differences in treatment, the overall survival rates were similar in the three regions. Adjustment for treatment in such a descriptive study may be misleading since different therapeutic strategies in different regions may lead to selection of patients of systematically better or poorer prognosis in the various treatment groups.


### Differences in treatment and survival rates of non-small-cell lung cancer in three regions of France

P Grosclaude[1], JP Galat[2], J Macé-Lesech[3], M Roumagnac-Machelard[1,4], M Mercier[2] and J Robillard[3]

[1]*Registre des cancers du Tarn, chemin des trois Tarn, 81000 Albi, France;* [2]*Registre des tumeurs du Doubs, CHR J Minjoz, 1 Bvd. Fleming, 25030 Besançon Cedex, France;* [3]*Registre des tumeurs du Calvados, Centre François Baclesse, Route de Lion sur Mer, 14021, Caen Cedex, France;* [4]*Centre Claudius Regaud, 20–24 rue du pont St Pierre, 31052 Toulouse Cedex, France.*

**Summary** Treatment and survival rates of patients with non-small-cell lung cancer (NSCLC) were compared between three French Cancer Registries (Calvados, Doubs, Tarn). The methodological issues in such comparisons are discussed. The treatments for NSCLC differed between the regions: radiotherapy tended to be preferred in Calvados (73% *vs* 21.3% surgery), whereas surgery was more frequently employed in Doubs and Tarn (27.7% and 37% respectively). The percentage of cases receiving no therapeutic treatment ranged from 7.8% (Calvados) to 26% (Tarn). Despite the differences in treatment, the overall survival rates were similar in the three regions. Adjustment for treatment in such a descriptive study may be misleading since different therapeutic strategies in different regions may lead to selection of patients of systematically better or poorer prognosis in the various treatment groups.

**Keywords**: non-small-cell lung cancer; treatment; survival; population-based registry

Lung (bronchus) cancer ranks first for both mortality and morbidity in men in France (Hill *et al.*, 1989; Benhamou *et al.*, 1990). Although its incidence is low in women, it is increasing, and is likely to continue to do so in view of the increasing numbers of female smokers. The prognosis for this cancer is still extremely poor and progress in suitable therapies is slow. Although there is a lack of consensus on the treatment of primary bronchial cancer, certain rules are widely accepted. It is generally considered that 25–30% of non-small-cell lung cancer (NSCLC) can be treated by surgery, and that radiotherapy is beneficial in 30% of other cases with more advanced stages (Johnson *et al.*, 1990). Palliative thoracic radiotherapy is also employed to reduce the major symptoms caused by tumoral extension (MRC Lung Cancer Working Party, 1991). A survival benefit of chemotherapy has been demonstrated in a randomised trial (Rapp *et al.*, 1988), although the adverse effects of chemotherapy have tended to restrict its use in this application (Splinter, 1990). Practitioners still lack effective treatment for a large number of lung tumours, which accounts for the diversity of strategies and the decision to withhold therapeutic treatment in numerous cases.

Although hospital series have indicated an increase in therapeutic efficacy, this was not confirmed in a recent study from the Scottish Cancer Registry, which failed to observe any overall improvement in the prognosis of lung cancer over the last 25 years (Black *et al.*, 1993). The therapeutic progress observed in hospital series may be attributed to the selection of the patients who are given the potentially more efficacious therapy. Population-based data are therefore of particular interest, although they do pose problems of comparability. In the present study we analysed data from three population-based cancer registries in an attempt to discern differences in treatment and overall survival from lung cancer in three distinct regions of France.

### Materials and methods

Out of the five general population-based cancer registries in France, three, Calvados in the north-west, Doubs in the east and Tarn in the south, possessed data relevant to this study. These regions also differ in their accessibility to health care. These three registries made available to us all cases of primary NSCLC diagnosed during the period 1987–88 for Doubs and Tarn, and in 1987 for Calvados. The present study included 615 cases (141 in Calvados, 274 in Doubs and 200 in Tarn). Age, gender histological type of tumour and treatment were recorded, but extent of disease or staging were not available. None of these three registries use autopsy reports as a data source. Under French legislation the death certificate cannot be employed as a data source, so there are no cases registered by death certificate only (DCO) in our study. The status (alive/dead) of all cases was ascertained on 31 December 1989. The information was collected by post from the department of vital statistics at the patient's place of birth. If the place of birth was not known, the patient's own doctor was contacted for the relevant information. Status data were obtained for 98% of the cases.

The age at diagnosis was known for all patients, and was split into three age ranges (<55 years, 55–74 years, >75 years) for the statistical analysis. A total of 97.8% of the cases had benefited from a pathological or cytological examination, and the result was coded according to the ICD-O classification (WHO, 1976). For the purpose of analysis, the tumours were grouped into four large categories: epidermoid carcinoma (ICD-O codes 80703, 80713, 80723, 80733), adenocarcinoma (ICD-O codes 81403, 81903, 82003, 82113, 82503, 82603, 83103, 84813), other documented pathological types (ICD-O codes 80123, 80203, 80213, 80313, 82403, 85603) and a fourth category including imprecise (ICD-O codes 80003, 80013, 80103) and absence of pathological/cytological examination. The treatment was coded in terms of three binary variables: surgery, radiotherapy and chemotherapy; radical and palliative treatment were not distinguished. The treatments received were known and were verified in each case, apart from two patients for whom the chemotherapy data were unavailable.

The distributions of tumour type, gender and age in the different regions were analysed using the chi-square test. The proportions of treated patients by age, gender, tumour morphology and regions were analysed by logistic regression. Survival univariate analysis was made using actuarial methods. The Cox model was employed for the multivariate survival analysis and the assumption of proportional hazards was checked graphically. The analyses were carried out using BMDP statistical software (F4, LR, 1L, 2L; Dixon, 1992).

Correspondence: P Grosclaude
Received 18 April 1994; revised 21 June 1995; accepted 22 June 1995.

### Results

*General characteristics (Table I)*

Differences in age distribution were noted between the three regions with younger cases in Calvados and older ones in Tarn. This was only partly due to the age structure of the population in the region, as for the period 1982–87 (Parkin *et al*., 1992) the incidence rate in young patients was higher in Calvados and Doubs than in Tarn. There was also a different sex distribution. Females represented 13.9% of the cases in Doubs compared with 8.5% in Calvados and 6% in Tarn ($P<0.05$), although this only partially accounts for the differences observed in the distribution of the histological types. Doubs was characterised by an overrepresentation of adenocarcinoma. Epidermoid cancer was more frequent in Tarn. The difference in pathological type between the regions ($P>0.01$) was still observed after adjustment for age ($P>0.01$) and sex ($P>0.01$).

*Treatment (Table II)*

Overall radiotherapy is used in 50.9% of the patients, chemotherapy in 30.3% and surgery in 29.3%. A total of 22.4% of the patients were treated by a combination of two treatments and 3% by a combination of three treatments; 18.2% received no therapeutic treatment. These figures however mask considerable differences between regions. In Calvados 73% of the cases received radiotherapy (as sole treatment in two out of three cases), whereas less than half of the cases in the other two regions benefited from radiotherapy. Surgery was more frequently employed in Tarn (37%) than in Doubs (27.7%) or Calvados (21.3%). Chemotherapy was used most in the Doubs region (39.1% of the cases), and the proportion of cases receiving no therapeutic treatment ranged from 7.8% in Calvados to 26% in Tarn. The percentage of cases not receiving therapeutic treatment was higher for those lacking accurate pathological data. This percentage also was found to increase with age.

Marked interregional differences emerged on analysis of the therapeutic treatments by logistic regression, even after taking the effects of age, gender and morphology into account (Table III). The results were in agreement with those of the univariate analysis. Surgery was more frequently employed in Tarn than in the other regions. Therapeutic treatment was also withheld more frequently in the Tarn region. Radiotherapy was more often used in Calvados, while chemotherapy was more frequently employed in Doubs than in the other two regions.

*Survival*

A univariate analysis of survival 18 months after diagnosis (Table IV) did not show any significant difference between regions (29.8% in Calvados, 30.0% in Tarn, 34.8% in Doubs) even after the effects of age or gender were taken into account. However, age and surgical treatment were correlated with prognosis. There was no difference in survival rates between the morphological groups, except for the cases with imprecise pathological results who had a lower survival rate. In view of the regional disparity in different variables (age, sex and pathological type), a multivariate analysis using

**Table I** General characteristics in each region

| | *Calvados (%)* | *Doubs (%)* | *Tarn (%)* | *Total (%)* |
|---|---|---|---|---|
| Age (years) | | | | |
| <55 | 25 (17.7) | 51 (18.6) | 19 (9.5) | 95 (15.4) |
| 55–74 | 97 (68.8) | 164 (59.9) | 131 (65.5) | 392 (63.7) |
| >75 | 19 (13.5) | 59 (21.3) | 50 (25.0) | 128 (20.8) |
| Sex | | | | |
| Male | 129 (91.5) | 236 (86.1) | 188 (94.0) | 553 (89.9) |
| Female | 12 (8.5) | 38 (13.9) | 12 (6.0) | 62 (10.1) |
| Morphology | | | | |
| Epidermoid | 78 (55.3) | 175 (63.9) | 140 (70.0) | 393 (63.9) |
| Adenocarcinoma | 25 (17.7) | 74 (27.0) | 39 (19.5) | 138 (22.4) |
| Other | 22 (15.6) | 2 (0.7) | 8 (4.0) | 32 (5.2) |
| NOS carcinoma | 16 (11.3) | 23 (8.4) | 13 (6.5) | 52 (8.5) |
| Total | 141 | 274 | 200 | 615 |

**Table II** Treatment according to age, sex, morphology and region

| | *Surgery (%)* | *Radiotherapy (%)* | *Chemotherapy (%)* | *No treatment (%)* |
|---|---|---|---|---|
| Age (years) | | | | |
| <55 | 35 (36.8) | 54 (56.8) | 42 (44.2) | 6 (6.3) |
| 55–74 | 136 (34.7) | 201 (51.2) | 126 (32.1) | 57 (14.5) |
| ⩾75 | 9 (7.0) | 58 (45.8) | 18 (14.1) | 49 (38.3) |
| Sex | | | | |
| Male | 157 (28.4) | 282 (50.1) | 163 (29.3) | 103 (18.5) |
| Female | 23 (37.1) | 31 (50.0) | 23 (37.1) | 9 (14.5) |
| Morphology | | | | |
| Epidermoid | 118 (30.0) | 208 (52.9) | 114 (29.0) | 67 (17.0) |
| Adenocarcinoma | 50 (36.2) | 66 (47.8) | 44 (31.9) | 24 (17.4) |
| Other | 7 (21.9) | 16 (50.0) | 12 (37.5) | 5 (15.6) |
| NOS carcinoma | 5 (9.6) | 23 (44.2) | 16 (30.8) | 16 (30.8) |
| Region | | | | |
| Calvados | 30 (21.3) | 103 (73.0) | 27 (19.3) | 11 (7.8) |
| Doubs | 76 (27.7) | 131 (47.8) | 107 (39.1) | 49 (17.9) |
| Tarn | 74 (37.0) | 79 (39.5) | 52 (26.0) | 52 (26.0) |
| Total | 180 | 313 | 186 | 112 |

**Table III** Multivariate analysis: differences in the treatment between age, sex, morphology and region

| | *Surgery* | | | *Radiotherapy* | | | *Chemotherapy* | | | *No treatment* | | |
|---|---|---|---|---|---|---|---|---|---|---|---|---|
| | *OR*[a] | *Standard error* | P-*value*[b] | *OR*[a] | *Standard error* | P-*value*[b] | *OR*[a] | *Standard error* | P-*value*[b] | *OR*[a] | *Standard error* | P-*value*[b] |
| Age (years) | | | | | | | | | | | | |
| $<55$ | 1.20 | 0.247 | NS | 1.23 | 0.240 | NS | 1.60 | 0.241 | NS | 0.41 | 0.450 | $P<0.05$ |
| 55–74[c] | 1.00 | | | 1.00 | | | 1.00 | | | 1.00 | | |
| $\geqslant 75$ | 0.13 | 0.366 | $P<0.001$ | 0.88 | 0.214 | NS | 0.31 | 0.283 | $P<0.001$ | 3.36 | 0.237 | $P<0.001$ |
| Sex | | | | | | | | | | | | |
| Male | 0.72 | 0.305 | NS | 1.00 | 0.288 | NS | 0.83 | 0.303 | NS | 1.26 | 0.410 | NS |
| Female[c] | 1.00 | | | 1.00 | | | 1.00 | | | 1.00 | | |
| Morphology | | | | | | | | | | | | |
| Epidermoid[c] | 1.00 | | | 1.00 | | | 1.00 | | | 1.00 | | |
| Adenocarcinoma | 1.25 | 0.226 | NS | 0.79 | 0.211 | NS | 0.98 | 0.230 | NS | 1.21 | 0.282 | NS |
| Other | 0.63 | 0.468 | NS | 0.43 | 0.414 | $P<0.05$ | 2.21 | 0.419 | NS | 1.88 | 0.564 | NS |
| NOS carcinoma | 0.32 | 0.495 | $P<0.05$ | 0.58 | 0.317 | NS | 1.36 | 0.342 | NS | 2.21 | 0.364 | $P<0.05$ |
| Region | | | | | | | | | | | | |
| Calvados | 0.41 | 0.305 | $P<0.001$ | 4.66 | 0.252 | $P<0.001$ | 0.49 | 0.288 | $P<0.05$ | 0.25 | 0.370 | $P<0.001$ |
| Doubs | 0.56 | 0.270 | $P<0.01$ | 1.38 | 0.193 | NS | 1.74 | 0.212 | $P<0.01$ | 0.66 | 0.240 | NS |
| Tarn[c] | 1.00 | | | 1.00 | | | 1.00 | | | 1.00 | | |

[a]Odds ratios for the treatment probabilities in categories compared with the reference category. [b]*P*-value for coefficients/standard error. [c]Reference category. NS, not significant, $P>0.05$.

the Cox model was carried out. No difference in survival between regions was found after adjustment (Table V). Age remained a significant risk factor, and significantly lower survivals were observed for the adenocarcinomas and for the cases with imprecise pathological results than for the other morphological types.

### Discussion

This analysis was carried out under the auspices of Eurocare, a concerned association of European population-based cancer registries (Berrino *et al.*, 1995). Its objective is to obtain information on the treatment and survival rates of all patients with lung cancer in the population. This study thus avoids the selection bias deriving from use of hospital series, and was designed to provide baseline information for assessment of the overall treatment of cancer patients by the health system. The data were obtained from complementary information on all the cases recorded by the relevant cancer registries over a given period. Various outside sources of information were consulted for each case to make the study as exhaustive as possible. In an attempt to obtain high quality data, we deliberately omitted data on the clinical stage of the tumour, and only collected information pertaining to treatment. Reliable data on the clinical stage are often not available, and the relatively high proportion of hospital records with no mention of the clinical stage has also been noted in a recent English study (Gulliford *et al.*, 1993). An American study showed that this absence was even more frequent for lung cancer than for other cancers, such as those of breast or colon (Feigl *et al.*, 1988). Although this does have some influence on the interpretation of our results, we thought it unlikely that there would be large differences in the distribution of the clinical stages at the time of diagnosis between the three regions.

The observed distribution of pathological types is consistent with that seen in other populations. Among lung cancers, the proportion of squamous cell carcinoma ranges from 45% to 50% and that of adenocarcinoma from 16% to 25% in the different European registries (Lutz *et al.*, 1988; Registre Genevois des Tumeurs, 1989; Zanetti *et al.*, 1992). The variations in the proportions of adenocarcinomas can be explained by the inclusion in the lung cancer category of different proportions of cases with primary or doubtful secondary tumours.

**Table IV** Survival of non-small-cell lung cancer (95% confidence intervals in parentheses)

| | *Survival at 18 months (%)* | *Log rank* $\chi^2$ | P-*value* |
|---|---|---|---|
| Sex | | | |
| Male | 31.7 (27.7–35.9) | 0.096 | NS |
| Female | 34.0 (23.0–47.0) | | |
| Age (years) | | | |
| $<55$ | 40.1 (30.3–50.7) | | |
| 55–74 | 33.8 (29.1–38.9) | 19.6 | $P<0.01$ |
| $>75$ | 19.6 (13.2–28.0) | | |
| Region | | | |
| Calvados | 29.8 (22.9–37.8) | | |
| Doubs - | 34.8 (28.8–41.3) | 0.3 | NS |
| Tarn | 30.0 (24.0–38.6) | | |
| Morphology | | | |
| Epidermoid | 33.3 (28.6–38.4) | | |
| Adenocarcinoma | 31.7 (24.1–40.3) | 12.8 | $P<0.01$ |
| Other | 37.5 (22.9–54.7) | | |
| NOS carcinoma | 18.3 (10.0–31.3) | | |
| Treatment | | | |
| Surgery | 65.8 (58.1–72.8) | | |
| Other | 18.6 (14.1–23.6) | 139.6 | $P<0.001$ |
| No treatment | 16.1 (10.3–24.3) | | |

NS, not significant, $P>0.05$.

**Table V** Factors associated with survival at 18 months of non-small-cell lung cancer: multivariate analysis

| | *Hazard ratio*[a] | *Standard error* | P-*value*[b] |
|---|---|---|---|
| Age (years) | | | |
| $<55$ | 0.71 | 0.146 | $P<0.01$ |
| 55–74[c] | 1.00 | | |
| $\geqslant 75$ | 1.58 | 0.118 | $P<0.01$ |
| Sex | | | |
| Male | 1.13 | 0.168 | NS |
| Female[c] | 1.00 | | |
| Morphology | | | |
| Epidermoid[c] | 1.00 | | |
| Adenocarcinoma | 1.28 | 0.124 | $P<0.05$ |
| Other | 0.92 | 0.216 | NS |
| NOS carcinoma | 1.6 | 0.164 | $P<0.01$ |
| Region | | | |
| Calvados | 1.13 | 0.128 | NS |
| Doubs | 0.98 | 0.114 | NS |
| Tarn[c] | 1.00 | | |

[a]Hazard ratios in categories compared with the reference category. [b]*P*-value for coefficients/standard error. [c]Reference category. NS, not significant, $P>0.05$.

There was a marked regional difference in treatment, which also emerged from a previous study on diagnostic attitude and regular treatment of lung cancer in 70 hospital teams (Mazover *et al.*, 1989). In our study, the differences in therapies between regions cannot be accounted for by differences in age, gender and morphology. The largest differences in treatment were observed between Calvados and Tarn, with Doubs being in the intermediate position. Radiotherapy was frequently employed in Calvados, often as sole treatment and in relatively few cases in combination with surgery. This contrasted with the situation in Tarn, where NSCLCs were generally treated surgically. The difference in therapeutic strategy has an influence on the number of non-treated patients. The fact that radiotherapy has fewer contraindications than surgery means that it can be employed on a larger proportion of patients, especially the elderly, and may account for the smaller proportion of untreated patients in Calvados compared with Tarn. The regional differences in treatment strategies may be explained by differences in the availability of services. For example, Calvados has a comprehensive cancer centre where radiotherapy is one of its specialties, whereas in Tarn many patients are treated in private clinics lacking specialised services.

There have been few detailed studies on lung cancer survival based on population data (Watkin *et al.*, 1990; Sant *et al.*, 1992). Most of the data on pathological types and treatments come from hospital data and the results of therapeutic trials, which are not readily extrapolated to the whole population of patients in a given region.

A more favourable prognosis for women has been noted by some cancer registries (Registre Genevois des Tumeurs, 1989; National Cancer Institute, 1991), although this has not been reported by other authors (McDuffie *et al.*, 1991; Sant *et al.*, 1992). To our knowledge, the poor prognosis of adenocarcinoma found in the present study has not been reported before. This illustrates the lack of comparability between the survival results of clinical trials and those obtained from population-based cancer registry data. The patients participating in clinical trials usually have complete records indicating the primary nature of the tumour, whereas the cancer registry data may include a number of secondary adenocarcinomas. These cancers, which are already metastatic, do not have a good prognosis and thus reduce the mean survival rate of the whole group.

The higher mortality of the older patients observed in our study is in line with the results of the study of the SEER Program comparing the relative survival of patients of different ages at the same clinical stage (Kant *et al.*, 1992).

Despite the differences in treatment, especially in the use of surgery, there was no overall difference in survival rates between the three regions. The prognostic value of surgery differed from one region to another, and its effectiveness appeared to be higher in the regions where it was least used. The greater use of surgery in Tarn is explained by the choice of surgical treatment for patients with an a priori poor prognosis. The prognosis for both surgically and non-surgically treated patients was thus worse in Tarn than in Calvados (Figure 1). For this reason, the introduction of this variable (surgery *vs* no surgery) in the model may be misleading (Table VI, models 1 and 2), since the assignment of patients to treatments did not imply the same a priori prognosis in the different region. The adjustment for surgery would therefore suggest that survival is significantly better in Doubs and in Calvados than in Tarn where a priori prognosis is worse than the two other populations, both for surgically treated patients and non-surgically treated patients. This phenomenon is similar to that described by Feinstein for stage migration, and has been referred to as the Will Rogers Phenomenon (Feinstein *et al.*, 1985). Treatment-adjusted analysis can erroneously predict a higher survival in areas (or groups) where the indications are the most selective, whereas the results for the overall population are identical in both areas.

### Conclusion

This study confirms the poor prognosis of lung cancer and the marked difference between patients who benefit from surgery and those who do not. We found a considerable diversity in the treatments offered between regions, and differences in the percentage of cases receiving no therapeutic treatment. These differences had no influence on survival rate, however, which was similar in the three regions. These observations lend support to the idea that prognostic

**Figure 1** Survival of non-small-cell lung cancer in relation to treatment. Sugery: □, Calvados; O, Tarn; Δ Doubs. No surgery: ▲, Doubs; ■, Calvados; ●, Tarn.

**Table VI** Multivariate analysis, adjustment on treatment

| | Model 1 | | | Model 2 | | |
|---|---|---|---|---|---|---|
| | *Hazard ratio*[a] | *Standard error* | P-*value*[b] | *Hazard ratio*[a] | *Standard error* | P-*value*[b] |
| Age (years) | | | | | | |
| <55 | 0.71 | 0.146 | $P<0.01$ | 0.81 | 0.146 | NS |
| 55–74[c] | 1.00 | | | 1.00 | | |
| ≥75 | 1.58 | 0.118 | $P<0.01$ | 1.06 | 0.119 | NS |
| Region | | | | | | |
| Calvados | 1.13 | 0.128 | NS | 0.83 | 0.130 | NS |
| Doubs | 0.98 | 0.114 | NS | 0.73 | 0.116 | $P<0.01$ |
| Tarn[c] | 1.00 | | | 1.00 | | |
| Treatment | | | | | | |
| Surgery | | | | 0.21 | 0.139 | $P<0.001$ |
| No surgery[c] | | | | 1.00 | | |

Analysis adjusted on sex and morphology. [a]Hazard ratios in categories compared with the reference category. [b]$P$-value for coefficients/standard error. [c]Reference category. NS, not significant, $P>0.05$.

differences between hospital series can be accounted for in terms of differences in the selection of patients. This highlights the utility of population data for an exact representation of lung cancer treatments, but also shows the inherent limitations of our data. To interpret the data in more detail and identify selection effects, registries need to record more comprehensive data, especially the clinical stage of the tumour at diagnosis. This will in turn require a closer collaboration with the relevant clinicians as this information is often not included in the hospital records.

**Acknowledgements**
We thank Dr J Estève for his valuable comments on a preliminary version of this paper. We would also like to thank Mrs M Gill and Drs A Desclaux and B Faliu for their technial assistance. The study was conducted under the auspices of the Network of French Registries (Francim) and was supported in part by a grant from INSERM, the Direction Générale de la Santé and the Ligue National Contre le Cancer.

**References**

BENHAMOU E, LAPLANCHE A, WARTELLE M, FAIVRE J, GIGNOUX M, MENEGOZ F, ROBILLARD J, SCHAFFER P, SCHRAUB S AND FLAMANT R. (1990). *Incidence des cancers en France 1978–1982.* Statistiques de Santé, INSERM: Paris.

BERRINO F, SANT M, VERDECCHIA A, CAPOCACCIA R, HAKULINEN T AND ESTEVE J (eds). (1995). *Survival of Cancer Patients in Europe: The Eurocare Study.* IARC scientific publications no. 132. IARC: Lyon.

BLACK JR, SHARP L AND KENDRICK SW. (1993). *Trends in Cancer Survival in Scotland 1968–1990.* Information and Statistics Division, National Health Service in Scotland: Edinburgh.

DIXON WJ (ed.). (1992). *BMDP Statistical Software.* Department of Biomathematics, University California Press: Los Angeles.

FIEGL P, GLAEFK G, FORD L, DIEHR P AND CHU J. (1988). Studying patterns of cancer care: how useful is the medical record? *Am. J. Public Health*, **78**, 526–533.

FEINSTEIN AR, SOSIN DM AND WELLS CK. (1985). The Will Rogers phenomenon. Stage migration and new diagnostic techniques as a source of misleading statistics for survival in cancer. *N. Engl. J. Med.*, **25**, 1604–1609.

GULLIFORD MC, BELL J, BOURNE HM AND PETRUCKEVITCH A. (1993). The reliability of cancer registry records. *Br. J. Cancer*, **67**, 819–821.

HILL C, BENHAMOU E, DOYON F AND FLAMANT R. (1989). *Evolution de la Mortalité par Cancer en France 1950–1985.* Statistiques de Santé, INSERM: Paris.

JOHNSON DH, EINHORN LH, BARTOLUCCI A, BIRCH R, OMURA G, PEREZ CA AND GRECO A. (1990). Thoracic radiotherapy does not prolong survival in patients with locally advanced, unresectable non small cell lung cancer. *Ann. Intern. Med.*, **113**, 33–38.

KANT AK, CLOVER C, HORM J, SCHATZKIN A AND HARRIS TB. (1992). Does cancer survival differ for older patients? *Cancer*, **70**, 2734–2740.

LUTZ JM, MENEGOZ F AND COLONNA M. (1988). *Le Cancer dans l'Isère 1979–1984.* Registre des cancers de l'Isère: Grenoble.

MCDUFFIE HH, KLAASSEN DJ AND DOSMAN JA. (1991). Men, women and primary lung cancer: a Saskatchewan personal interview study. *J. Clin. Epidemiol.*, **6**, 537–544.

MAZOYER G AND GUERIN JC. (1989). Le bilan pré-thérapeutique du cancer bronchique primitif. *Rev. Pneumo. Clin.*, **45**, 23–27.

MEDICAL RESEARCH COUNCIL LUNG CANCER WORKING PARTY. (1991). Inoperable non-small-cell lung cancer (NSCLC): a Medical Research Council randomised trial of palliative radiotherapy with two fractions or ten fractions. *Br. J. Cancer*, **63**, 265–270.

NATIONAL CANCER INSTITUTE. (1991). Surveillance, Epidemiolology, and End Results (SEER) Program. Division of cancer prevention and control. Cancer statistic review 1973–88. US Department of Health and human services: NIH Publication no. 91-2789: Bethesda.

PARKIN D, MUIR C, WHELAN S, GAO Y, FERLAY J AND POWELS J. (eds). (1992). *Cancer Incidence in Five Continents.* Vol. VI, IARC scientific publications no. 120. IARC: Lyon.

RAPP E, PATER JL, WILLAN A, CORMIER Y, MURRAY N, EVANS WK, IAN HODSON D, CLARK DA, FELD R, ARNOLD AM, AYOUB JI, WILSON KS, LATREILLE J, WIERZBICKI RF AND HILL DP. (1988). Chemotherapy can prolong survival in patients with advanced non small-cell lung cancer – Report of Canadian multicenter randomized trial. *J. Clin. Oncol.*, **6**, 633–641.

REGISTRE GENEVOIS DES TUMEURS. (1989). *Cancer à Genève, Incidence, Mortalité, Survie, 1970–1986.* Registre Genevois Des Tumeurs: Geneva.

SANT M, GATTA G, CAPOCACCIA R, VERDECCHIA A, MICHELI A, SPECIALE D, PASTORINO U AND BERRINO F. (1992). Survival for lung cancer in northern Italy. *Cancer Causes & Control*, **3**, 223–230.

SPLINTER TAW. (1990). Chemotherapy in advanced non small cell-lung cancer. *Eur. J. Cancer*, **10**, 1093–1099.

WATKIN SW, HAYHURST GK AND GREEN JA. (1990). Time trends in the outcome of lung cancer management: a study of 9090 cases diagnosed in the Mersey Region 1974–1986. *Br. J. Cancer*, **61**, 590–596.

WORLD HEALTH ORGANIZATION. (1976). *International Classification of Diseases for Oncology*, first edn. WHO: Geneva.

ZANETTI R AND CROSIGNANI P (eds). (1992). *Il cancro in Italia, i Dati di Incidenza dei Registri Tumori 1983–1987.* Lega Italiana per la Lotta contro i Tumori, Associazione Italiana de Epidemiologia: Torino.